# Collegial Ethics: Supporting Our Colleagues

**DOI:** 10.1007/s11948-012-9364-9

**Published:** 2012-04-26

**Authors:** Michael J. Kuhar, Dorthie Cross

**Affiliations:** 1 Candler Professor of Neuropharmacology, Georgia Research Alliance Eminent Scholar, School of Medicine, Yerkes National Primate Research Center of Emory University, 954 Gatewood Rd, NE, Atlanta, GA 30329 USA; 2 Center for Ethics of Emory University, Atlanta, GA USA; 3 The Department of Psychology, Emory University, Druid Hills, GA USA

**Keywords:** Collegial ethics, Colleagues, Supporting colleagues, Do no harm

## Abstract

The goal of collegial ethics is to actively support our colleagues and to develop the skills needed to do so. While collegial interactions are key for our careers, there is little or no training in this. Many of our actions and reactions with our colleagues are instinctive. Human nature has evolved to be self-protective, but many evolved and automatic responses to others are not always in the best interests of our society or of us. Developing courage and a style of supportive language, avoiding destructive acts, and adhering to the golden rule will improve our relationships and provide a more positive environment for all.

## Collegial Behavior

How do we act towards our colleagues? Many of us would answer that we should be pleasant and cordial. But this essay suggests that we go further, that we *actively* support our colleagues—even in troubled times. This assumes that the colleague is supportable which may not always be the case. In everyday life there are many opportunities to support our coworkers, and a good reason for doing this is that it enhances the quality of our own lives as well. When things are going smoothly, and there are no conflicts or problems, it can be relatively easy to offer support. Wishing others well and helping where we can will be appreciated. But, what about when there is trouble? Most of us have known a colleague in trouble or in crisis. The problem may be a personal failure, an external attack, or perhaps a complex turn in one’s professional career. The trouble can be serious such as an accusation of misconduct or blacklisting (Kuhar 2008, 2009), or any one of many, less serious problems that befall us that are still distressing. Unfortunately, when serious accusations are made, damage to the accused can persist in some form, even if the accused is vindicated (Lubalin and Matheson 1999).

Seeing colleagues in trouble, we ask ourselves if we should get involved, and how much effort we should make. This essay briefly addresses the questions of when and why should we help our colleagues? Note that this is specifically *not* about how to accuse, address or prosecute faculty who get into trouble; this is about how to *support* faculty, even those who are injured or in some sort of peril or conflict, if it is appropriate. Because these situations take on ethical issues, this topic is called “collegial ethics” (Kuhar 2011). A compilation of all methods and approaches for supporting colleagues would take up volumes, but we will address a few that we have experienced and discussed over the years. We’re writing this because, like many, we know of those who have been victimized by circumstances and by shortcomings in human nature. We also feel that timely help and effective collegial support would have made a difference, at least in some cases. The setting for this essay is academia, but it, of course, applies to any community or group. It also summarizes and extends an earlier communication (Kuhar 2011).

It may not be easy to help a colleague. Sometimes we are not sure what the facts are, or sometimes we lack the structure to channel our involvement. We also know that taking a stand on a controversial issue can be perilous and hurt our career (Bird and Hoffman-Kim 1998); therefore we may perceive a danger to ourselves and decide to stay out of it. An easy but sometimes questionable way to avoid involvement is to simply blame the victim or the accused! We often hear, “He got himself into it, let him get himself out of it! He can do it himself! He’s always in trouble. He’s always been controversial.” Yet another way to skirt the issue is to invoke excessive fairness; it may be obvious that someone has been wronged, but you avoid taking sides and stay in the neutral middle by saying you want “to be fair.” Taking such positions makes it easy to ignore the situation and to avoid self examination and action. But maybe a more ethical view, and one that is even more self-benefiting in the long run, is to be attentive and ask ourselves if we have a stake in the situation. What will happen if we do not get involved? What would we want others to do for us? Justice and compassion may rely on intervention and leadership by others.

If a crime has been committed by a colleague then support may not be appropriate. But the law is not perfect. It says that one is not guilty until proven guilty and legal guilt is for the courts to decide. But there is a difference between legal guilt and ethical guilt. Legally, we are “not guilty” until proven otherwise, but if ethically guilty (you really did it), then there should be some remedy. Even if we cannot prove legal guilt, an ethical debt may have been incurred.

## How Do We Help Our Colleagues?

How do we approach helping and supporting our colleagues? What are the rules or guidelines that we can use? While there are not yet formal guidelines, several seem commendable. Of course, common sense cannot be ignored. Time and resources are limited and we cannot get involved in every issue, but it is important to ask how any given situation impacts us, our colleagues, and our institutions? When are the losses or gains great enough that we must step up? How much should we contribute just because we are fellow human beings?

There are several additional factors to consider. There is the golden rule which is found in all or most societies (Runes 1959): help others as we would like to be helped. This seems like a no brainer, but in fact, the golden rule might never even reach our awareness, particularly in the heat of a conflict. Self righteousness and judgment, perhaps based in fear, often prevail. While judgment maybe needed, it seems that fairness and compassion are needed as well.

We can also support a colleague by using appropriate language and communication. We have to be careful about how we *talk* about a given situation or colleagues (Kuhar 2011). Unfortunately, the language used in conversations about hurt or accused colleagues is often not helpful and is often more like judgmental “gossip” or posturing than thoughtful discussion. Hurtful things can be done and said in a casual setting without realizing their impact and influence on others. We need to develop guidelines for these situations, and we need to develop a style and a language of mutual support among our colleagues.

## Careful What You Say—You May End Up Believing It

Consider the following fictitious situation. You are a young colleague, perhaps an assistant professor, having lunch with several other faculty members. They have known each other for years, and you are glad to be there because they represent the “establishment” in the department. All of you are having a discussion about another younger colleague who is not present. There is a rumor that he, the absent younger colleague, has inappropriately modified some of his research findings, but no one seems to know the real facts of the case, and the colleague has not been interviewed or spoken to by anyone present.

One of the more senior members of the group says:

“We’ve talked about this before and this behavior can’t be tolerated. We need to deal with this ethical issue quickly, and I prefer, harshly.”

Another member of the group agrees.

“I’ve been suspicious of him for some time for other reasons. We need to come down hard on him.”

Others, nod their heads in agreement. But, they notice that you have not agreed, and they look at you inquisitively. You really do not agree with them. So, do you nod in agreement—bowing to pressure to do so? Or, do you point out that the real facts are not yet clear to you? Do you point out that because he is younger, newer, and not as well known as some other colleagues, caution is needed for sake of fairness? Do you say that we shouldn’t speak of him as guilty, but that the situation is under examination? This is a predicament. Do you take the easy way out by nodding in agreement, or do you find some courage and state your real opinions, which might annoy the group? Of course, embracing collegial ethics would mean remaining cautious and open until the facts are certain, and honestly expressing your concern about rushing to judgment.

Agreeing to something you really do not believe creates an internal dissonance, and the data may surprise you. Experimental studies have found that after you agree to something you really do not privately believe, your private opinion changes to bring it more in line with what you have said (Festinger and Carlsmith 1959; Gawronski and Strack 2004). Just because you *state* agreement, not because you see more evidence or logic, you move your real, personal position towards agreement! You can at least partly convince yourself just by *saying* something, even if it is under only mild pressure.

If we truly want to be fair to ourselves and our colleagues, then we need to withhold stating false opinions and speak the truth of our opinions. Again, courage is a major factor in what we might do. We often know what the “right” thing to do is, but we may not do it. Our instincts are to preserve our own safety, and these are very strong urges, honed by eons of evolution. We often decide just to go along and get along and mind our own business. But how do we gather and promote the *courage*—and it seems to be often a matter of *courage*—to stand up or speak up and be supportive of others. Probably courage can be managed just like any other personality trait or behavior. Gathering support among colleagues and friends is especially effective in reducing the fear of acting and being alone. It can help you see in advance the kinds of risk you may be taking and the range of possible fallout. Discussions with others can help clarify the issues when they are complex and help us sort out the best point of view. Even without social support, courageous actions can still be carried out. It is recognized that courage does not imply that there is no fear. Mark Twain said that “courage is resistance to fear, mastery of fear—not absence of fear” (Twain 2010). In the opinion of many, courage in supportive and ethical actions has been underestimated.

## Survival Instincts and Judgment of Others

It goes without saying that our very human nature drives much of our behavior. We react to our colleagues according to social norms and inherited responses that have evolved to protect the species. We avoid those who are somehow stigmatized by illness (communicable pathogens?), appearances (physical disabilities reflective of illnesses?), odd behavior (dangerous to others?) or some other marker (being exiled?) (Kurzban and Leary 2001; Allen and Babcock 2003, 2006; Park et al. 2003; Gilbert 1998). We have evolved to make automatic judgments about situations and people to protect ourselves and our groups, and although these judgments may generally help us, we run the risk of causing harm if we judge too quickly. A central part of collegial ethics is: one, that these norms and evolved automatic responses be recognized as only norms and responses, and two, that they be examined to see if complying with them is helpful in all situations. Consider the colleague from the earlier example. While it is understandable that we may want to distance ourselves from someone accused of academic dishonesty, we risk harming another’s career without knowing enough to make a fair judgment. In another example, our innate response to people with physical disabilities may be to avoid them, but they may have much to offer us if we help them overcome or adapt to their disability. Someone with a broken leg or depression may be dysfunctional, without appeal, and therefore avoided. But in this day and age, they may be restored to society by treatment. In cognitive therapy, a successful form of psychotherapy, there is a focus on “correcting” a variety of “errors and distortions” in thinking and reasoning (Gilbert 1998). Our immediate, evolved responses and patterns of thinking may not always serve us or others well in the long run.

## First Do No Harm—or Minimize Harm

Another useful guideline in dealing with colleagues derives from the Hippocratic Oath—first do no harm. Consider a fictitious case (all of the included cases, while reminiscent of real occurrences, are not drawn from any specific event). A new faculty member, Dr Sharpe, is in a kind of trouble. He is outstanding in many ways: he is very successful, comes from a high powered University, and is a recent “trophy” hire. But, many of the other faculty members are critical of him, perhaps because he has been critical of their productivity (It turns out that his boss, the Director, has been critical of the same faculty members). They are beginning to harp on his flaws, and negative rumors that he is overly judgmental and hostile are becoming vicious, even though is little evidence to support the rumors. Even though Sharpe has denied the truth of the rumors, they have hurt Sharpe’s reputation, and invitations for seminars and lectures have fallen off significantly. Dr Sharpe has asked for support from colleagues and superiors, but it has not helped. Some members of the faculty and the administration think it would be best if he left the school.

The Director who hired Dr Sharpe is somewhat overwhelmed by the trouble, and is unsure of what he should do. He does not think the rumors about his new hire are true, but he will not even admit that rumors exist when Sharpe asks about them. Nonetheless, it is clear to Sharpe that there are problems, perhaps serious ones. He repeatedly goes to his Director for help and clarification without really getting any.

The Director, who is new and not very experienced himself, has been thinking about his options. He has been urged by his superiors to find the courage to do *something*.

1. The Director can ask Dr Sharpe to leave. Maybe he can dig into his past and reframe earlier problems in a negative way so that Sharpe is too embarrassed to stay. This seems easy, allows the Director to largely ignore the problem, and shifts the burden to Dr Sharpe to get out of the way. But some of his friends, more experienced administrators, point out that this will be harmful to Sharpe because it will appear to outsiders that he could not be defended, and was undesirable.
2. He can try to get another department or school interested in Sharpe. The Director knows many department heads throughout the country and is considering calling them to say that Sharpe is “available” for hire. He actually does this with one other chair, but is met with suspicion and a blunt “no.” The other chair has heard about the rumors and wants to know if they are true and if Sharpe is in real trouble. He asks if the Director is trying to pass on his problem to someone else. The Director begins to realize that he may be hurting Sharpe by taking this option.
3. The Director can explain the situation to Dr Sharpe and work with him and his opponents towards a solution. This will take time and effort, but will show that he has faith in Sharpe, and that Sharpe still has value. But it will also mean that he may have to stand up against those who want Sharpe out of the school. The Director decides to announce that he plans to discuss this with Sharpe, find out the facts, and bring in professional helpers, if needed, to resolve the situation to everyone’s satisfaction as best he can. Even though the Director may be in some peril with this option, he feels that it is his responsibility.

The last option is obviously the collegial solution. Even though he may end up investigating Dr Sharpe for bad behavior, it does give Sharpe the dignity and due process he deserves. It may be that Sharpe will decide to leave anyway, or perhaps everyone will be satisfied by a to-be-discovered solution. The Director is to be congratulated in that he chose to avoid the solutions that were easy but actually did harm to Dr Sharpe. The guideline of doing no harm may be especially useful when, for one reason or another, we don’t know the facts and cannot easily discover them. You may say, with some validity, that life is not so simple. No matter what we do we may harm someone in some way. If a situation does involve some harm no matter what, then minimizing harm is obviously the goal.

## Not a Trivial Issue

Some may feel that this whole topic of collegial ethics is almost a trivial issue. But consider that over recent decades, there have been remarkable advances in ethics in related areas such as the protection of animal and human subjects in research. Ethical training in those areas required that we spend time with related precedents, cases, rules and guidelines. We regularly review those rules and guidelines and update ourselves (PHS 2010; NIH 2010). This raises our awareness of ethical behavior in research and increases correct action. Do we need a similar practice in “collegial ethics”? If we spent time with case histories, guidelines, precedents and the development of supportive skills, might that help us deal with colleagues and make our institutions a better place? Implementing this type of approach in collegial ethics seems very advantageous. However, collegial ethics is rarely formally addressed in scientific training such as in Ph.D. programs. Instead ethics training focuses, rightly, on the protection of patients and participants but with little consideration of our colleagues. We are trained to identify possible ethical transgressions, but are left not knowing how to approach the implicated colleagues. What to do is addressed in the situation where we think a colleague has done something wrong, but is it addressed in a more positive way, where we actively offer support to colleagues in a variety of situations? Probably not. One could even take the view that there is a serious need for the development of personal/collegial ethics. Omar Bradley, a highly successful World War II General, said “The world has achieved brilliance without wisdom, power without conscience. Ours is a world of nuclear giants and ethical infants” (Bradley 1967). Effective training and practice of collegial ethics is still very much in its infancy.

There are examples where colleagues have successfully grouped together for mutual support. Women in science, for example, have organized to promote needed policy changes (AWIS 2010). Also, administrative bodies have promoted policies that offer support. For example, universities have established offices for the benefit of post doctoral trainees (Office of Post Doctoral Education 2010), a group often underrepresented in the past. So, things have been done and progress has been made, but we need to go further.

## Conclusions

What are the things that we as individuals can do to develop collegial ethics, and how do we do it? Well, we need to be aware if things we say or do are *destructive* to others in some way. If they are, we need to think twice about it, even though we may not be able to avoid it. As noted above, the golden rule is a useful guideline, and the mandate of the Hippocratic Oath—first do no harm—is useful as well. Also, we need to develop a neutral or supportive language when talking about problems and colleagues. Related to this, developing skills in communication is helpful in all aspects of career development. Conflict resolution is a practice that helps us find solutions to conflicts, at least in many cases (Conflict Resolution 2010; Communication 2010; Communication Matters 2010). Developing habits of win–win thinking (What is Win–Win 2010; Covey 2010) applies here as well and helps us look at the bigger picture that includes the other party. It also provides a sense of optimism that a mutually compatible solution can be found. And, because conflicts can be due to a variety of differences, we need to cherish diversity rather than be suspicious of it (Battaini-Dragoni 2010; Lahn and Ebenstein 2009). These are the kinds of skills that we could nurture and develop over many years with great benefit. Using actions, compassion, fairness, and courage in the pursuit of a supportive position with colleagues is worth stressing, and further development of this topic seems needed.
